# Intestinal Dysbiosis, Tight Junction Proteins, and Inflammation in Rheumatoid Arthritis Patients: A Cross-Sectional Study

**DOI:** 10.3390/ijms25168649

**Published:** 2024-08-08

**Authors:** Arkaitz Mucientes, José Manuel Lisbona-Montañez, Natalia Mena-Vázquez, Patricia Ruiz-Limón, Sara Manrique-Arija, Aimara García-Studer, Fernando Ortiz-Márquez, Antonio Fernández-Nebro

**Affiliations:** 1 Instituto de Investigación Biomédica de Málaga y Plataforma en Nanomedicina-IBIMA Plataforma Bionand, 29010 Málaga, Spain; arkaitz.mucientes@ibima.eu (A.M.); josemanuellisbona@hotmail.com (J.M.L.-M.); patrilimon@hotmail.com (P.R.-L.); sarama_82@hotmail.com (S.M.-A.); aimara.garcia95@gmail.com (A.G.-S.); fomquez@gmail.com (F.O.-M.); afnebro@gmail.com (A.F.-N.); 2 UGC de Reumatología, Hospital Regional Universitario de Málaga, 29009 Málaga, Spain; 3 Departamento de Medicina y Dermatología, Universidad de Málaga, 29010 Málaga, Spain; 4 Department of Endocrinology and Nutrition, Virgen de la Victoria University Hospital, 29010 Málaga, Spain; 5 CIBER in Physiopathology of Obesity and Nutrition (CIBEROBN), Carlos III Health Institute, 28029 Madrid, Spain

**Keywords:** tight junction proteins, intestinal permeability, gut microbiota, rheumatoid arthritis

## Abstract

Recent studies point to intestinal permeability as an important factor in the establishment and development of rheumatoid arthritis (RA). Tight junctions (TJs) play a major role in intestinal homeostasis. The alteration of this homeostasis is related to RA. Furthermore, RA patients present dysbiosis and a lower microbiota diversity compared to healthy individuals. A cross-sectional study including RA patients and sex- and age-matched healthy controls was performed. The quantification of TJ proteins was carried out by ELISA. Gut microbiota was evaluated by NGS platform Ion Torrent S. The inflammatory variables included were DAS28, CRP, inflammatory cytokines (IL-6, IL-1, TNF-α) and oxidised LDL. Claudin-1 levels showed significant differences between groups. Results evidenced a correlation between claudin-1 values and age (*r*: −0.293; *p* < 0.05), IL6 (*r*: −0.290; *p* < 0.05) and CRP (*r*: −0.327; *p* < 0.05), and between zonulin values and both age (*r*: 0.267; *p* < 0.05) and TNFα (*r*: 0.266; *p* < 0.05). Moreover, claudin-1 and CRP levels are related in RA patients (*β*: −0.619; *p*: 0.045), and in patients with high inflammatory activity, the abundance of the genus *Veillonella* is positively associated with claudin-1 levels (*β*: 39.000; *p*: 0.004).

## 1. Introduction

Rheumatoid arthritis (RA) stands as a complex autoimmune condition characterised by chronic inflammation primarily affecting the joints. With a global prevalence of approximately 1%, RA is a major health burden [1]. The pathology of RA involves a dysregulated immune response, leading to synovial inflammation, joint damage, and systemic complications [2]. Despite the research effort, the exact aetiology of RA remains unknown. The aetiology of RA is considered to be multifactorial, involving a complex interaction of genetic predisposition and environmental factors [3,4,5].

**Figure 1:**
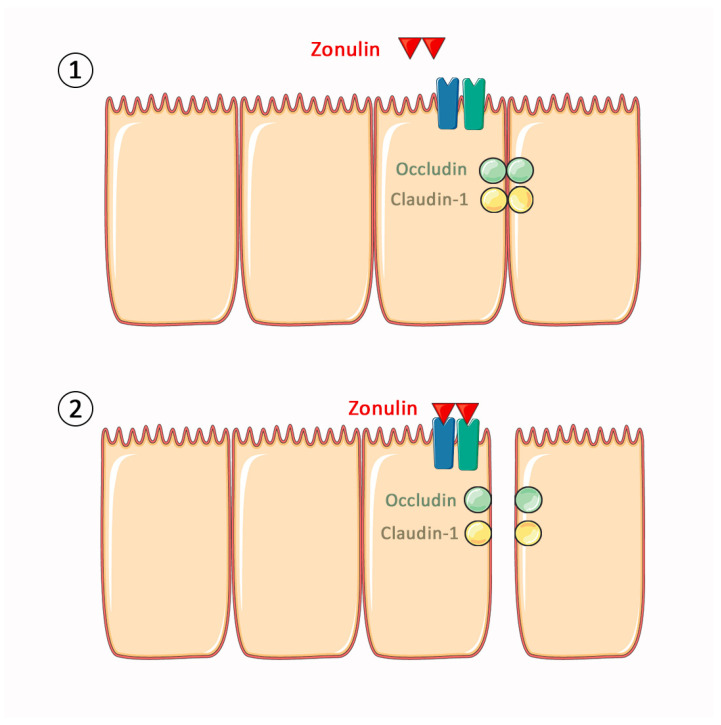
Schematic representation of the gastrointestinal barrier, highlighting the tight junction (TJ) proteins analysed. Zonulin (red) modulates intestinal permeability by regulating TJ disassembly. Occludin (green) and claudin-1 (yellow) are structural proteins of the TJ that co-ordinately maintain the integrity and selective permeability of the barrier. When zonulin binds the EGFR (dark blue) and PAR2 (dark green) receptors, it activates a cascade of reactions that ultimately result in the displacement of occludin and claudin-1, leading to loosening of the TJs (2). When zonulin signalling stops, the TJs return to their closed basal state (1).

Intestinal permeability, a crucial aspect of gastrointestinal physiology, has gained attention for its potential implications in various systemic inflammatory conditions, including autoimmune disorders such as RA [6,7,8]. Although RA patients are likely to present with a higher intestinal permeability, only a few studies have succeeded in making conclusions about intestinal permeability [9,10]. Thus, elevated intestinal permeability in RA patients remains to be further investigated. Among the various players responsible for intestinal permeability, the tight junctions (TJs) stand out, whose function is regulated by several factors of two routes [11]. TJs are composed of a complex network of proteins. In terms of their function in the TJs, the transmembrane proteins occludin (65 KDa) and claudin-1 (20–27 KDa) are structural components of the TJs, while zonulin, a ~47 Kda protein secreted by epithelial cells, is a potent regulator of the TJs [12,13,14]. Therefore, these proteins play pivotal roles in maintaining the integrity of the intestinal barrier. Figure 1 resumes the role and the existing interrelation of the three proteins. Investigating the relationship between these proteins and RA may provide insights into the underlying mechanisms linking the gut and systemic inflammation.

Despite the growing interest in the potential link between intestinal permeability and RA, the existing literature lacks conclusive evidence. Previous studies have described that increased epithelial permeability in RA patients is likely to lead to oral and intestinal microbiota products influencing the pathogenesis and disease activity [15,16]. Nevertheless, these studies analysed serum proteins rather than stool proteins despite the fact that stool samples may directly reflect the intestinal permeability, not being influenced by the systemic circulation [17,18].

Another modifying factor of intestinal permeability is the gut microbiota. The relationship between intestinal barrier function and gut microbiota has been communicated [7]. Previous studies have established that RA patients present dysbiosis in different grades and a lower microbiota diversity compared to healthy individuals [19,20,21]. Furthermore, it is thought that intestinal dysbiosis is associated with clinical characteristics of RA, such as immunoglobulin levels or rheumatoid factor [22]. Although the relationship between microbiota and RA has received increasing interest, most published studies are observational studies in which differences in the composition of the microbiota between patients and healthy individuals have been analysed. Therefore, the existing literature regarding the mechanism underlying the dysbiosis observed in RA patients is lacking.

Hence, our study aims to explore intestinal permeability in stool from both RA patients and healthy controls (HCs) by the quantitative analysis of three key tight junction proteins—occludin, claudin-1, and zonulin. Then, the relationship between the levels of TJ proteins and the abundance of the different taxa composing the microbiota of RA patients will be analysed. Through this research, we seek to address the existing knowledge gaps and contribute valuable insights that may inform future therapeutic strategies targeting the gut–microbiota–joint axis.

## 2. Results

### 2.1. Cohort

A total of 164 subjects were included in the study: 82 RA patients and 82 HCs. Table 1 shows the main clinical and demographic characteristics of the cohort. Most of the patients were women (76%) with a mean age of around 56 years. There was no difference according to age, sex, and race between both groups. However, patients with RA had both higher smoking habits (53.7% vs. 39.0%, *p* = 0.028) and obesity defined by WHO (35.4% vs. 22.0%, *p* = 0.057) than HCs, while in the remaining comorbidities, there were no differences between groups. Additionally, more than half of RA patients had erosive disease, 80% tested positive for autoantibodies, and all were on disease-modifying antirheumatic drug (DMARD) treatment.

Table 2 summarises the inflammatory characteristics of the included individuals. RA patients also showed higher levels of acute phase reactants erythrocyte sedimentation rate (ESR), C-reactive protein (CRP), interleukin levels (IL-6, IL-1β, TNFα), homocysteine, and leukocytes than HCs, while they displayed lower physical activity levels and presented lower haemoglobin levels than HCs. Moreover, there were no differences between groups regarding Mediterranean diet adherence, adipocytokines, insulin-like growth factor 1 (IGF-1), and lipid levels. A total of 52 patients (63.4%) had maintained a mean Disease Activity Score-28 with Erythrocyte Sedimentation Rate (DAS28-ESR) of low inflammatory activity (<3.2) and 30 (36.6%) of moderate or high activity (≥3.2) throughout their follow-up.

### 2.2. Tight Junction Proteins Quantification by ELISA

**Figure 2:**
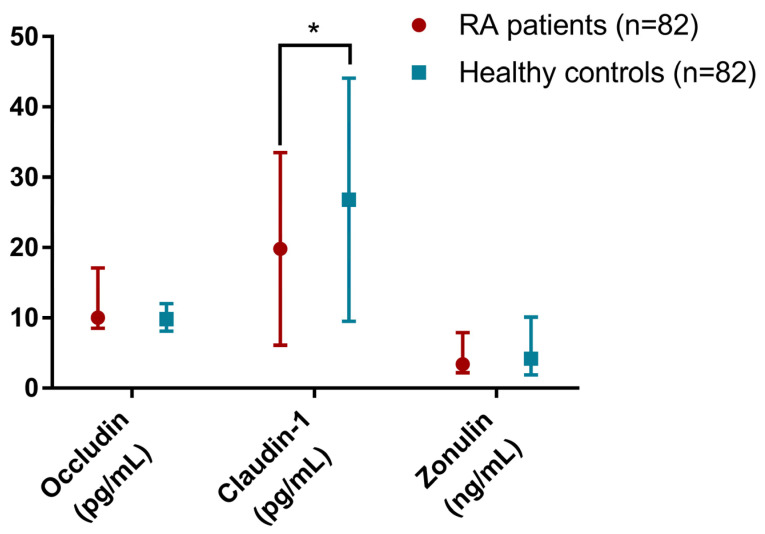
Concentration of the distinct tight junction proteins analysed in faeces. Red: rheumatoid arthritis (RA) patients; blue: healthy controls. Data are shown as mean ± standard deviation for claudin-1 and median (p25–p75) for occludin and zonulin. Significance level: * *p* ≤ 0.05.

The presence of the three proteins in stool was quantified, revealing compelling results (Figure 2). Claudin-1 levels were significantly lower in RA patients than in HCs (mean: 19.8, SD: 13.7 vs. mean: 26.8, SD: 17.3, *p* = 0.024). Conversely, neither occludin (median: 10.0, IQR: 8.5–17.1 vs. median: 9.8, IQR: 8.1–12.0, *p* = 0.412) nor zonulin (median: 3.4, IQR: 2.2–7.9 vs. median: 4.2, IQR: 1.9–10.1, *p* = 0.431) showed significant differences between groups.

### 2.3. Gut Microbiota Association Study

The results of the association analysis between TJ protein levels and taxa abundance detected in RA patients showed a positive correlation between occludin levels and the abundance of genus *rc4_4* (*r* = 0.241, *p* = 0.022) of an unknown family of order *RF32* (*r* = 0.222, *p* = 0.036) and of an unknown genus of order *RF32* (*r* = 0.222, *p* = 0.036). Likewise, a positive correlation was observed between claudin-1 levels and the abundance of genus *Veillonella* (*r* = 0.272, *p* = 0.033), and there was no correlation between zonulin levels and the abundance of any taxa (Table S1). Classifying RA patients according to inflammatory activity, defined by DAS28-ESR with cut-off point 3.2, claudin-1 levels showed no correlation with the abundance of any taxa in low-disease-activity patients, while in high-disease-activity patients, claudin-1 levels correlated with the abundance of an unknown genus of the family *Lachnospiraceae* (*r* = 0.615, *p* ≤ 0.01) and the genus *Veillonella* (*r* = 0.858, *p* ≤ 0.01) (Table S2).

### 2.4. Biomarkers

The calculated Pearson’s correlation coefficients are shown in Table 3. Occludin exhibits a significant negative correlation with average HAQ (*r*= −0.271, *p* = 0.044). Additionally, claudin-1 demonstrates correlations with age (*r* = −0.293, *p* = 0.037), IL-6 (*r* = −0.290, *p* = 0.040), and CRP (*r* = −0.327, *p* = 0.039), while zonulin correlates positively with age (*r* = 0.267, *p* = 0.018) and TNFα (*r* = 0.266, *p* = 0.024).

The results of linear regression models evaluating whether RA characteristics are associated with claudin-1 levels in the cohort, for both HC and RA patients, are shown in Table 4. The analysis established a negative association between CRP and claudin-1 levels (*p* = 0.048). Table 5 shows the results of both univariant and multivariant linear regression models investigating factors associated with claudin-1 levels in RA patients. It determined that CRP showed a significant negative association with claudin-1 levels in multivariate analyses (*p* = 0.045). Furthermore, when the multivariate analysis was restricted to the subgroup of patients with RA who had not achieved clinical remission, i.e., DAS28-ESR ≥ 3.2, the abundance of the genus *Veillonella* is positively associated with claudin-1 levels (*p* = 0.004) (Table 6).

## 3. Discussion

The intestinal epithelial barrier plays a key role in the control of the balance between tolerance and immunity to non-self-antigens since this barrier separates the internal milieu from the immune cells [23]. Defects in its integrity result in immune cell activation and chronic inflammation [24]. The integrity of the intestinal barrier depends on several factors. Among these factors, tight junctions (TJ) are crucial in the integrity of the barrier and regulate the exchange of fluids, molecules, and leukocytes between the intestinal lumen and the bloodstream. Therefore, TJs are part of the system protecting the body against foreign antigens and microorganisms. Thus, malfunction of the TJ is likely to be a possible cause in autoimmune conditions in which altered intestinal permeability is described, such as RA [8,25]. Another important factor affecting the integrity of the intestinal barrier is the microbiota. Dysbiosis is commonly defined as an imbalance in the gut microbiota, often associated with diseases such as rheumatoid arthritis (RA) [20]. Dysbiosis is characterised by an increased intestinal permeability [26], so a balanced microbiota is required for a correct intestinal barrier function. Due to its inner interactions with the intestinal barrier, a potential feedback mechanism between the intestinal barrier and the microbiota has been suggested [27]. Despite several studies addressing the dysbiosis–intestinal permeability axis [27,28,29,30], studies exploring whether TJs play a role in the mechanisms underlying intestinal permeability are scarce. Understanding the role of TJ proteins in RA aetiology and whether they are related to the dysbiosis described in RA patients is relevant for developing targeted therapeutic interventions and improving patient outcomes.

Most studies analysing TJs and their regulation to assess intestinal permeability in distinct pathologies have used serum samples. However, we decided to use stool samples in order to avoid possible interferences in the results since the studied proteins are expressed in other tissues besides the bowel [31,32,33].

The quantification of TJ proteins in stool showed that RA patients presented a lower concentration of claudin-1 compared to HCs, whereas there were no significant differences between groups regarding occludin and zonulin. Claudins are a family of transmembrane proteins with sizes from 20 to 27 kDa and with 27 members that are identified in humans and expressed in a tissue-specific manner [34,35]. Among the family members, claudin-1 was analysed since it is the only member expressed along the entire gastrointestinal tract [36]. Additionally, claudin-1 has been associated with several gastrointestinal diseases, including inflammatory conditions such as ulcerative colitis and Crohn’s disease [37]. In line with our results, Tajik et al. reported that RA patients showed lower claudin-1 RNA levels, as well as a decreased presence of claudin-1 in ileal mucosal biopsies compared to HCs [38]. The barrier function conducted by TJs is based on two pathways: the pore pathway and the leak pathway [39]. These pathways are regulated independently, so the decrease of proteins responsible for one pathway does not affect the other pathway [11,40]. Considering that the pore pathway is regulated by claudin family proteins [39], our results suggest that the decrease in claudin-1 may be involved in RA pathology by increasing intestinal permeability as a result of a malfunction of the pore pathway. Occludin is also a transmembrane protein, with a size of 65 KDa, and it presents a 90% homology among mammals [33]. Interestingly, despite being a structural component of the TJs, it has been demonstrated in murine models that the absence of occludin does not affect the morphology of the TJs [41]. Moreover, several post-transcriptional and post-translational modifications affecting occludin function and, thus, intestinal permeability have been reported [33]. It is known that occludin expression is subject to gene splicing events that result in different isoforms of the protein [42]. Among them, the occludin isoform generated by skipping the exon 4 has been related to TJ ‘loosening’ [43]. Furthermore, the occludin phosphorylation level has gained attention as a factor related to barrier and TJ regulation, and several studies have addressed whether its phosphorylation status is linked to barrier dysfunction [44,45,46,47,48]. Although our results showed no significant differences between groups after quantifying occludin, it remains possible that this protein is involved in the increased permeability described in RA patients since ELISA assays do not detect protein modifications. Specific studies are required to determine whether RA patients present a modified occludin related to a malfunction of the TJ, which would result in increased gut permeability.

The failure of the intestinal barrier favours a pro-inflammatory environment [49]. Given that correlation analysis showed that claudin-1 stool levels inversely correlate with both IL-6 and C-reactive protein (CRP) serum levels, our data are consistent with this statement since IL-6 is a marker of systemic inflammation, while CRP, stimulated by IL-6 [50], is a routine inflammation marker in RA [51,52]. Moreover, the downregulation of claudin-1 has been associated with the inflammatory process in atopic dermatitis and various tumours [53,54,55,56]. Furthermore, zonulin, a modulator of TJs whose increase enhances intestinal permeability [13], shows a positive correlation with tumour necrosis factor-alpha (TNFα), a pro-inflammatory cytokine related to RA and recently proposed as a biomarker for RA activity [57,58]. Occludin, a structural component of TJs whose exact role in barrier function is unclear [38], inversely correlates with HAQ score, suggesting that its reduction increases functional disability. Our correlation results support these proteins being evaluated as biomarkers in the RA context in further studies. Finally, the multiple linear regression carried out evidenced the existing inverse relation between stool levels of claudin-1 and serum levels of CRP. This association would confirm that a decrease in claudin-1 levels, resulting in TJ looseness, could underlie the inflammation characteristic of RA.

Currently, there is a consensus that RA patients have a lower abundance and diversity in their microbiota than healthy subjects [59]. Our group carried out a descriptive comparative study between the microbiota of RA patients and healthy subjects, in which the existence of taxa specifically associated with RA was reported [60]. Since the origin of this dysbiosis is unknown, we decided to study whether there was any relationship between RA-associated taxa and the presence of occludin, claudin-1, and zonulin. In our results, zonulin showed no correlation with any taxa, whereas claudin correlated with an unknown family and genus of the order RF32, related to the response to anti-TNFα treatment in inflammatory bowel disease patients [61] and with the genus rc4_4, related to high-fat diet-induced obesity in animal models [62]. Claudin-1 levels showed a positive correlation with the genus *Veillonella*, which has been described as elevated in saliva and the synovial fluid of RA patients compared to HCs and osteoarthritis patients, respectively [63,64]. Interestingly, when studying the correlations by classifying patients according to disease activity, it was observed that in the low-activity group, there was no correlation between claudin-1 and any taxa, while in the high-activity patients, the correlation with *Veillonella* and an unknown genus of the family *Lachnospiraceae* reported to associate with inflammatory bowel disease [65]. Taken together, our results suggest that claudin-1 and occludin could be somehow involved in the underlying processes of dysbiosis in RA. However, since TJs are complex structures involving more proteins than those studied, such as other members of the claudins or zonula occludens-1, the structural proteins of TJs [39], it cannot be ruled out that some of these proteins may also be related to RA and its processes.

The present study has several limitations that need to be acknowledged. First, the sample size of the patient group with high inflammatory activity was limited, which limited the analysis of the relationship between claudin-1 levels and RA-associated characteristics. Second, there is a racial bias, as nearly all individuals in the cohort are Caucasian, which may limit the generalisability of our findings to more diverse populations. Third, the cross-sectional design of our study prevents any inference of causality between the levels of the studied proteins and the development or progression of intestinal permeability and dysbiosis in RA patients. The analysis of plasma levels of lipopolysaccharide-binding protein (LBP) would be a useful approach for the assessment of intestinal permeability [66]. Longitudinal studies would be beneficial in establishing a temporal relationship. Fourth, ELISA assays were used to quantify the protein levels, which, while informative, do not provide insights into the functional activity of these proteins or their interactions with gut microbiota, which would provide a deeper understanding of their role in RA pathogenesis. Future studies should include larger, more diverse cohorts and employ techniques that can assess the functional dynamics of these proteins to further elucidate their role in RA pathogenesis.

In summary, our results suggest a relevant role of claudin-1 in RA, mainly in the inflammation process. Moreover, our data suggest that claudin-1 and occludin, structural proteins of TJs, may be related to dysbiosis in RA patients. Further research is warranted to elucidate the precise role of claudin-1 in the context of RA and to explore both its clinical implications and its potential relevance as a biomarker for distinguishing healthy individuals from those with RA.

## 4. Materials and Methods

### 4.1. Cohort/Study Design

A cross-sectional study including rheumatoid arthritis (RA) patients and sex- and age-matched healthy controls (HC) was performed. RA patients were classified according to the 2010 criteria of the American College of Rheumatology/European League against Rheumatism [67]. The exclusion criteria were the presence of inflammatory or rheumatic diseases other than RA (except for secondary Sjögren’s syndrome), diabetes, or any non-controlled general condition. Furthermore, individuals with extreme diets, those exposed to antibiotic therapy (current or previous 3 months), those taking probiotic agents, and those who had started any new treatment were also excluded. HCs were not relatives of the patients but were recruited from the same geographical area as the patients. The faeces of individuals were collected during routine rheumatologist visits.

All subjects gave their informed consent for inclusion before they participated in the study. The study was conducted in accordance with the Declaration of Helsinki, and the protocol was approved by the Ethics Committee of Málaga (“Comité de Ética de la Investigación de Málaga”, identification code: 4/2016, PI9).

### 4.2. Clinical and Laboratory Variables

Patients were assessed using a standardised clinical interview and clinical analysis before enrollment. Demographic, clinical, laboratory, and treatment-related data were recorded by a rheumatologist. Inflammatory mediators such as TNF-α, IL-1β, IL-6, and IGF-I, as well as malondialdehyde-oxidised LDL, were measured in plasma using an enzyme-linked immunosorbent assay (ELISA) following the manufacturer’s instructions (TNF- α, QTA00C, R&D Systems Inc., Minneapolis, MN, USA; IL-1β, QLB00B, R&D Systems Inc.; IL-6, Q6000B, R&D Systems Inc.; IGF-I, WHO 02/254, Mediagnost Gmbh., Tuebingen, Germany; ox-LDL, BI-20032, Biomedica Gmbh., Wien, Austria). DAS28-ESR [68] and the Health Assessment Questionnaire (HAQ) [69] were estimated at baseline and during follow-up. The physical activity of the cohort was measured by the International Physical Activity Questionnaire (IPAQ) [70], defining sedentariness as an IPAQ score < 600. The adherence to the diet was assessed using the Mediterranean Diet Adherence Screener (MEDAS) questionnaire from the PREDIMED study [71]. Moderate-to-high activity was defined as a DAS28-ESR score of ≥3.2. Finally, diagnosis delay was defined as the time, in months, from the onset of the symptoms to the date of diagnosis.

### 4.3. Tight Junction Protein Quantification in Faeces

The quantification was carried out by ELISA. The preparation of the faeces samples for the assays consisted of shaking faeces with phosphate-buffered saline (PBS) (10 mg faeces to 100 μL PBS), centrifugation of the homogenate at 1000× *g* for 20 min, careful collection of the supernatant, and immediately assaying or cryopreserving the samples.

Specific commercial kits were used for each protein (Occludin, CSB-EL016263HU, CUSABIO Innovation Center, Houston, TX, USA; Claudin-1, CSB-EL005490HU, CUSABIO; and Zonulin, CSB-EQ027649HU, CUSABIO; https://www.cusabio.com/, (accessed on 1 January 2024) according to the manufacturer’s instructions and then measured in a plate reader (VERSAmax™ tunable, Molecular Devices LLC, San Jose, CA, USA).

### 4.4. Microbiota Data

To determine a possible relationship between the quantified proteins (occludin, claudin-1, and zonulin) and the abundance of the different taxa present in the microbiota of RA patients, we used data previously obtained in our group by 16S rRNA sequencing in the patients included in this study.

Briefly, the Ion 16 S Metagenomics Kit and Ion Plus Fragment Library Kit (Thermo Fisher Scientific, Inc., Waltham, MA, USA) were used to prepare the amplicon library. Emulsion PCR and sequencing of the amplicon libraries were performed on an Ion 530 chip (Ion 530^TM^ Chip Kit, Thermo Fisher Scientific Inc., Waltham, MA, USA) using the Ion Chef System and Torrent S5^TM^ system, respectively (Ion 510™/520™/530™ Kit-Chef, Thermo Fisher Scientific Inc., Waltham, MA, USA), following the manufacturer’s instructions. Finally, the bioinformatic analysis was conducted in order to perform taxonomic analysis, as previously described [60].

### 4.5. Statistical Analysis

A descriptive analysis was carried out based on absolute frequencies and percentages for the qualitative variables and mean (standard deviation) or median (interquartile range) for the quantitative variables. Whether the data follow the normal distribution was analysed by the Kolmogorov–Smirnov test.

Both groups, RA patients and HCs, were compared by Pearson’s χ^2^ test or *t*-test, as appropriate. In order to study the correlations between tight junction protein values and both RA clinical variables and microbiota data, the Pearson or Spearman correlation coefficient was calculated according to the normality of the variables. Finally, a multiple linear regression was carried out to identify claudin-1-related factors in the total cohort, RA patients, and high-activity RA patients; the variables included in the model were those that were significant in the bivariate analysis and of clinical interest. The statistical analyses were performed using IBM SPSS Statistics for Mac OS, Version 28 (IBM Corp., Armonk, NY, USA), licensed to the staff of the University of Malaga. The significance level was set at *p* < 0.05.

## 5. Conclusions

Our results point out that rheumatoid arthritis (RA) patients show differences in intestinal permeability compared to healthy controls due to decreasing claudin-1 levels. Moreover, C-reactive protein levels are inversely associated with claudin-1 levels in stool, which suggests a possible role of inflammation in claudin-1 regulation in RA. In addition, tight junction proteins in stool are correlated with RA clinical parameters, supporting a potential use as biomarkers in the RA context. Finally, our results suggest that claudin-1 and occludin could be a cause of the dysbiosis observed in RA patients.

## Figures and Tables

**Table 1:** Clinical and demographic characteristics of the cohort.

Variable	RA Patients (*n* = 82)	Healthy Controls (*n* = 82)	*p*-Value
Age (years), mean (SD)	56.3 (11.1)	56.3 (10.9)	0.986
Sex (woman), *n* (%)	63 (76.8)	63 (76.8)	1.000
Caucasian, *n* (%)	81 (98.8)	81 (98.8)	1.000
BMI (Kg/m^2^), mean (SD)	28.3 (5.3)	27.0 (4.2)	0.072
Smoking			0.028
Never, *n* (%)	35 (42.7)	50 (61.0)	
Ex-smoker, *n* (%)	20 (24.4)	9 (11.0)	
Smoker, *n* (%)	27 (32.9)	23 (28.0)	
Comorbidities			
Arterial hypertension, *n* (%)	25 (30.5)	20 (24.4)	0.382
Diabetes Mellitus, *n* (%)	6 (7.3)	2 (2.4)	0.147
Dyslipidaemia, *n* (%)	19 (23.2)	16 (19.5)	0.567
WHO obesity, *n* (%)	29 (35.4)	18 (22.0)	0.057
Clinic-analytic characteristics			
Evolution time (months), median (p25–p75)	91.3 (77.6–122.7)	-	-
Diagnosis delay (months), median (p25–p75)	8.1 (4.6–14.2)	-	-
Erosions, *n* (%)	52 (63.4)	-	-
RF > 10 U/mL, *n* (%)	66 (80.5)	0 (0.0)	<0.001
ACPA > 20 U/mL, *n* (%)	67 (81.7)	0 (0.0)	<0.001
Treatment			
Synthetic DMARDs, *n* (%)	75 (91.5)	-	-
Methotrexate, *n* (%)	59 (72.0)	0 (0.0)	<0.001
Leflunomide, *n* (%)	9 (11.0)	0 (0.0)	0.002
Sulfasalazine, *n* (%)	10 (12.2)	0 (0.0)	0.001
Hydroxychloroquine, *n* (%)	6 (7.3)	0 (0.0)	0.013
Biologic DMARDs, *n* (%)	27 (32.9)	0 (0.0)	<0.001
Anti-TNFα, *n* (%)	20 (24.4)	0 (0.0)	<0.001
Anti-IL-6, *n* (%)	6 (7.3)	0 (0.0)	0.013
Rituximab, *n* (%)	1 (1.2)	0 (0.0)	0.316
Glucocorticoid at protocol, *n* (%)	17 (20.7)	0 (0.0)	<0.001

RA: rheumatoid arthritis; BMI: body mass index; WHO: World Health Organization; RF: rheumatoid factor; ACPA: anti-citrullinated protein antibodies; SD: standard deviation; DMARD: disease-modifying antirheumatic drug.

**Table 2:** Inflammatory characteristics of the cohort.

Variable	RA Patients (*n* = 82)	Healthy Controls (*n* = 82)	*p*-Value
DAS28-ESR average value, mean (SD)	3.0 (0.7)	-	-
Remission-Low activity, *n* (%)	52 (63.4)	-	-
Moderate-High activity, *n* (%)	30 (36.6)	-	-
DAS28-ESR at index-date, mean (SD)	2.9 (1.0)	-	-
Remission-Low activity, *n* (%)	53 (64.6)	-	-
Moderate-High activity, *n* (%)	29 (35.4)	-	-
HAQ average value, mean (SD)	0.6 (0.5)	-	-
HAQ at index-date, mean (SD)	0.7 (0.6)	-	-
Laboratory parameters			
ESR (mm/h), median (IQR)	13 (7.2–21.0)	11.0 (6.0–16.0)	0.044
Haemoglobin (g/dL), median (p25–p75)	13.2 (12.3–14.4)	14.0 (12.8–14.9)	0.018
Leukocytes (10^9^/L), median (p25–p75)	6375.0 (5410.0–8102.5)	6040.0 (4980.0–7700.0)	0.014
Platelet (10^9^/L), mean (SD)	243,426.8 (64,859.3)	236,146.3 (55,291.8)	0.440
Total cholesterol (mg/dL), mean (SD)	202.1 (36.5)	209.5 (35.6)	0.188
LDL cholesterol (mg/dL), median (p25–p75)	115.9 (101.1–139.2)	123.5 (101.7–148.5)	0.150
HDL cholesterol (mg/dL), median (p25–p75)	58.0 (51.0–65.2)	61.5 (50.7–73.2)	0.202
Triglycerides (mg/dL), median (p25–p75)	89.5 (67.7–142.2)	86.5 (69.0–125.2)	0.699
Homocysteine (mg/dL), median (p25–p75)	14.1 (12.0–17.1)	11.6 (9.6–14.9)	0.001
Adipocytokines, lipoproteins, and interleukins			
Leptin (ng/mL), median (p25–p75)	15.5 (8.3–33.1)	19.1 (8.7–35.1)	0.628
Resistin (ng/mL), mean (SD)	7.6 (3.3)	7.8 (3.2)	0.817
Adiponectin (µg/mL), mean (SD)	12,104.5 (6228.4)	10,202.7 (6263.6)	0.118
IL-6 (pg/mL), median (p25–p75)	11.0 (5.3–23.3)	4.1 (2.7–5.7)	<0.001
CRP (mg/L), median (p25–p75)	3.4 (2.9–7.0)	2.9 (2.0–3.0)	<0.001
IL-1β (pg/mL), median (p25–p75)	4.3 (4.1–4.4)	2.8 (2.6–4.1)	<0.001
TNFα (pg/mL), median (p25–p75)	4.7 (3.5–11.1)	3.5 (3.0–4.4)	<0.001
IGF-1 (pg/mL), mean (SD)	164.8 (85.5–226.3)	139.1 (48.9–298.9)	0.462
LDL-oxidase (UI/mL), median (p25–p75)	2.5 (0.8–5.6)	1.2 (0.3–2.7)	0.065
Physical activity and Mediterranean diet			
IPAQ (METs), median (p25–p75)	334.5 (198.0–792.0)	647.0 (336.7–990.0)	0.002
Sedentariness, *n* (%)	54 (65.9)	38 (46.3)	0.042
MEDAS (≥9), *n* (%)	52 (63.4)	47 (57.3)	0.425

RA: rheumatoid arthritis; SD: standard deviation; IQR: interquartile range; DAS28: Disease Activity Score-28; HAQ: Health Assessment Questionnaire; CRP: C-reactive protein; ESR: erythrocyte sedimentation rate; IL-6: interleukin 6; IPAQ: International Physical Activity Questionnaire; METs: Metabolic Equivalent of Task units; MEDAS: Mediterranean Diet Adherence Score; LDL: low-density lipoprotein; HDL: high-density lipoprotein; IGF-1: insulin-like growth factor 1.

**Table 3:** Correlation between the level of tight junction proteins and RA characteristics.

Variable	Occludin (pg/mL)	Claudin-1 (pg/mL)	Zonulin (ng/mL)
Age (years)	−0.054	−0.293 *	0.267 *
BMI (Kg/m^2^)	−0.014	−0.177	0.093
Evolution (months)	0.127	−0.114	0.082
Diagnosis delay (months)	0.209	−0.121	0.152
RF (U/mL)	0.041	0.136	−0.080
ACPA (U/mL)	−0.026	−0.193	0.082
DAS28-ESR average	−0.143	−0.116	0.011
HAQ average	−0.271 *	−0.161	0.095
ESR (mm/h)	−0.032	−0.045	−0.048
Leptin (ng/mL)	−0.014	0.013	−0.113
Resistin (ng/mL)	−0.157	−0.285	0.101
Adiponectin (µg/mL)	−0.174	−0.151	−0.129
IL-6 (pg/mL)	0.038	−0.290 *	−0.129
CRP (mg/mL)	−0.141	−0.327 *	0.122
IL-1β (pg/mL)	0.019	0.190	0.181
TNFα (pg/mL)	0.055	0.092	0.266 *
IGF-1 (pg/mL)	0.120	−0.075	0.145
LDL-oxidase (UI/mL)	0.078	0.155	0.023
IPAQ (METs)	−0.018	−0.250	0.011

BMI: body mass index; RF: rheumatoid factor; ACPAs: anti-citrullinated protein antibodies: DAS-ESR: Disease Activity Score-28 with Erythrocyte Sedimentation Rate; HAQ: Health Assessment Questionnaire; ESR: erythrocyte sedimentation rate; CRP: C-reactive protein; IPAQ: International Physical Activity Questionnaire. LDL: low-density lipoprotein; IGF-1: insulin-like growth factor 1; IL-6: interleukin 6; IL-1β: interleukin 1β; TNFα: tumour necrosis factor α; METs: Metabolic Equivalent of Task units. Significance level: * *p* < 0.05.

**Table 4:** Linear regression model of claudin-1-related factors in total subjects.

Variable	B Univariant (CI95%)	B Multivariant (CI95%)	*p*-Value
Age (years)	0.172 (−0.117, 0.461)		
Sex (woman)	1.137 (−6.291, 8.565)		
CRP (mg/L)	−0.511 (−1.334, 0.312)	−2.690 (−5.370, −1.013)	0.048
Smoking	−0.675 (−0.905, 5.555)		
Obesity	0.567 (−3.395, 4.529)		
Genus *Veillonella*	6.001 (−0.480, 12.482)		
Family *Lachnospiraceae*	−9.136 (−2.048, −20.321)		

Naglekerke’s R^2^ = 0.109. Variables included in the multivariant regression model: age, sex, CRP, obesity, smoking, and genus Veillonella and family Lachnospiraceae. Abbreviations: CRP: C-reactive protein.

**Table 5:** Linear regression model of claudin-1-related factors in RA patients.

Variable	Univariant (CI95%)	Multivariant (CI95%)	*p*-Value
Age (years)	−0.396 (−0.767, −0.025)	−0.314 (−0.682, 0.055)	0.093
Sex (woman)	3.891 (−5.925, 13.706)		
CRP (mg/L)	−0.731 (−1.331, −0.130)	−0.619 (−1.222, −0.015)	0.045
ACPA+	−9.967 (−20.365, 0.432)		
Smoking	−4.306 (−12.280, 3.668)		
Obesity	0.758 (−4.003, 5.519)		
Genus *Veillonella*	7.542 (−0.633, 15.716)		
Family *Lachnospiraceae*	7.157 (−7.670, 21.984)		

Naglekerke’s R^2^ = 0.127. Variables included in the multivariant equation: age, sex, CRP, ACPA, obesity, smoking, and genus *Veillonella* and family *Lachnospiraceae*. Abbreviations: RA, rheumatoid arthritis; CRP, C-reactive protein; ACPA+, positive for anti-citrullinated peptide antibody.

**Table 6:** Linear regression model of claudin-1-related factors in RA patients with high inflammatory activity (n = 28).

Variable	Univariant (CI95%)	Multivariant (CI95%)	*p*-Value
Age (years)	0.283 (−0.662, 1.228)		
Sex (woman)	6.433 (−26.042, 38.906)		
CRP (mg/mL)	0.203 (−1.621, 1.214)		
ACPA+	5.676 (−17.211, 28.563)		
Smoking	−14.680 (−34.158, 4.797)		
Obesity	0.890 (−10.010, 7.120)		
Genus *Veillonella*	39.080 (14.658, 63.502)	39.000 (14.650, 63.600)	0.004
Family *Lachnospiraceae*	30.800 (−4.496, 66.096)		

Naglekerke’s R^2^ = 0.399. Variables included in the equation: age, sex, CRP, ACPA, obesity, smoking, and genus Veillonella and family Lachnospiraceae. Abbreviations: RA, rheumatoid arthritis; CRP, C-reactive protein; ACPA+, positive for anti-citrullinated peptide antibody.

## Data Availability

The original contributions presented in the study are included in the article/Supplementary Materials; further inquiries can be directed to the corresponding author/s.

## Supplementary Materials

The following supporting information can be downloaded at: https://www.mdpi.com/article/10.3390/ijms25168649/s1.
