# Supplementary material for: Intestinal Dysbiosis, Tight Junction Proteins, and Inflammation in Rheumatoid Arthritis Patients: A Cross-Sectional Study
Source: Int J Mol Sci. 2024 Aug 8;25(16):8649. doi: 10.3390/ijms25168649 (PMC11354395; doi:10.3390/ijms25168649)
Supplement: Supplementary file 1 [file ijms-25-08649-s001.zip › ijms-3117426-supplementary.pdf]

**Table S1.** Correlation between TJ proteins levels and the abundance, in ASVs, of the phyla detected in RA patients gut microbiota.

| **Gut microbiota** | **Occludin (pg/mL)** | **Claudin-1 (pg/mL)** | **Zonulin (ng/mL)** |
|---|---|---|---|
| *Actinobacteria* | -0.079 | -0.110 | 0.074 |
| *Firmicutes* | 0.123 | 0.081 | 0.131 |
| *Synergistetes* | -0.035 | -0.052 | 0.057 |
| *Coriobacteriaceae* | -0.110 | -0.125 | -0.093 |
| *Streptococcaceae* | 0.074 | 0.023 | 0.055 |
| *EtOH8* | 0.024 | 0.054 | -0.052 |
| Unknown family of order *RF32* | **0.222*** | 0.057 | -0.066 |
| *Oxalobacteraceae* | -0.115 | -0.054 | 0.188 |
| *Bifidobacterium* | -0.049 | -0.081 | 0.161 |
| *Collinsella* | -0.144 | -0.125 | -0.094 |
| *Alistipes* | -0.093 | -0.061 | 0.067 |
| *Weissella* | -0.087 | -0.096 | -0.054 |
| Unknown genus of family *EtOH8* | 0.024 | 0.054 | -0.052 |
| *Pseudoramibacter_Eubacterium* | -0.056 | -0.106 | 0.008 |
| Unknown genus of family *Lachnospiraceae* | 0.073 | 0.140 | -0.103 |
| *Coprococcus* | 0.008 | -0.118 | -0.144 |
| *Dorea* | -0.040 | -0.113 | -0.056 |
| *rc4_4* | **0.241*** | 0.193 | -0.013 |
| *Anaerotruncus* | -0.082 | 0.152 | -0.052 |
| *Veillonella* | 0.065 | **0.272*** | -0.088 |
| *Mogibacterium* | -0.073 | -0.095 | -0.076 |
| Unknown genus of family *Erysipelotrichaceae* | 0.025 | -0.128 | -0.046 |
| Unknown genus of order *RF32* | **0.222*** | 0.057 | -0.066 |
| *Succinatimonas* | -0.040 | -0.039 | -0.041 |
| *Synergistes* | -0.078 | 0.073 | 0.085 |
| Unknown genus of class *TM7-3* | -0.092 | -0.185 | -0.067 |

ASVs: amplicon sequence variants. Significance level: * $p<0.05$

**Table S2.** Correlation between claudin-1 levels and the abundance, in ASVs, of the phyla detected in RA patients according to inflammatory activity.

| Gut microbiota | Low inflammatory activity ($n$=53) | High inflammatory activity ($n$=29) |
|---|---|---|
| *Actinobacteria* | -0.175 | -0.003 |
| *Firmicutes* | 0.042 | 0.256 |
| *Synergistetes* | -0.062 | -0.036 |
| *Coriobacteriaceae* | -0.087 | 0.010 |
| *EtOH8* | 0.065 | 0.050 |
| Unknown family of order *RF32* | 0.045 | 0.051 |
| *Oxalobacteraceae* | -0.082 | -0.193 |
| *Bifidobacterium* | -0.178 | -0.001 |
| *Collinsella* | -0.065 | -0.015 |
| *Alistipes* | -0.265 | 0.052 |
| *Weissella* | 0.119 | -0.208 |
| *Pseudoramibacter_Eubacterium* | -0.258 | -0.211 |
| Unknown genus of family *Lachnospiraceae* | -0.031 | **0.615**** |
| *Coprococcus* | -0.076 | -0.152 |
| *Dorea* | -0.130 | 0.037 |
| *Anaerotruncus* | -0.139 | -0.033 |
| *Veillonella* | 0.048 | **0.858**** |
| *Mogibacterium* | -0.153 | -0.118 |
| Unknown genus of family *Erysipelotrichaceae* | -0.245 | 0.385 |
| Unknown genus of order *RF32* | 0.045 | 0.051 |
| *Synergistes* | 0.009 | 0.017 |
| Unknown genus of class *TM7-3* | -0.270 | 0.235 |

ASVs: amplicon sequence variants. Significance level: ** $p$<0.01
